# Crystal structure of raptor adenovirus 1 fibre head and role of the beta-hairpin in siadenovirus fibre head domains

**DOI:** 10.1186/s12985-016-0558-7

**Published:** 2016-06-22

**Authors:** Thanh H. Nguyen, Mónika Z. Ballmann, Huyen T. Do, Hai N. Truong, Mária Benkő, Balázs Harrach, Mark J. van Raaij

**Affiliations:** Departamento de Estructura de Macromoléculas, Centro Nacional de Biotecnología (CNB-CSIC), Calle Darwin 3, E-28049 Madrid, Spain; Genetic Engineering Laboratory, Institute of Biotechnology (IBT-VAST), 18 Hoang Quoc Viet, Cau Giay, Hanoi, Vietnam; Institute for Veterinary Medical Research, Centre for Agricultural Research, Hungarian Academy of Sciences, Budapest, Hungary

**Keywords:** Siadenovirus, Atomic structure, Beta-hairpin, Deletion mutagenesis, Protein stability, X-ray crystallography

## Abstract

**Background:**

Most adenoviruses recognize their host cells via an interaction of their fibre head domains with a primary receptor. The structural framework of adenovirus fibre heads is conserved between the different adenovirus genera for which crystal structures have been determined (*Mastadenovirus, Aviadenovirus, Atadenovirus* and *Siadenovirus*), but genus-specific differences have also been observed. The only known siadenovirus fibre head structure, that of turkey adenovirus 3 (TAdV-3), revealed a twisted beta-sandwich resembling the reovirus fibre head architecture more than that of other adenovirus fibre heads, plus a unique beta-hairpin embracing a neighbouring monomer. The TAdV-3 fibre head was shown to bind sialyllactose.

**Methods:**

Raptor adenovirus 1 (RAdV-1) fibre head was expressed, crystallized and its structure was solved and refined at 1.5 Å resolution. The structure could be solved by molecular replacement using the TAdV-3 fibre head structure as a search model, despite them sharing a sequence identity of only 19 %. Versions of both the RAdV-1 and TAdV-3 fibre heads with their beta-hairpin arm deleted were prepared and their stabilities were compared with the non-mutated proteins by a thermal unfolding assay.

**Results:**

The structure of the RAdV-1 fibre head contains the same twisted ABCJ-GHID beta-sandwich and beta-hairpin arm as the TAdV-3 fibre head. However, while the predicted electro-potential surface charge of the TAdV-3 fibre head is mainly positive, the RAdV-1 fibre head shows positively and negatively charged patches and does not appear to bind sialyllactose. Deletion of the beta-hairpin arm does not affect the structure of the raptor adenovirus 1 fibre head and only affects the stability of the RAdV-1 and TAdV-3 fibre heads slightly.

**Conclusions:**

The high-resolution structure of RAdV-1 fibre head is the second known structure of a siadenovirus fibre head domain. The structure shows that the siadenovirus fibre head structure is conserved, but differences in the predicted surface charge suggest that RAdV-1 uses a different natural receptor for cell attachment than TAdV-3. Deletion of the beta-hairpin arm shows little impact on the structure and stability of the siadenovirus fibre heads.

## Background

Adenoviruses were first discovered in human adenoid tissue in 1953 [1]. In humans, depending on the type, they associate with respiratory, ocular and gastrointestinal infections [2, 3]. At present, there are no approved anti-adenoviral drug therapies [4, 5]. On the other hand, adenovirus soon became an important model for studying fundamental molecular biology concepts and recombinant versions have found applications as gene transfer vectors, anti-cancer agents and vaccination vehicles [6–10]. Five distinct adenovirus genera have been identified: *Mastadenovirus* (infecting mammals and including all human adenoviruses), *Aviadenovirus* (infecting birds), *Atadenovirus* (infecting snakes, lizards, birds and ruminants), *Siadenovirus* (infecting birds, frog and tortoise) and *Ichtadenovirus* (infecting fish) [11–13].

Adenoviruses are non-enveloped viruses with a linear, double-stranded DNA genome, packed into an icosahedral capsid with a vertex-to-vertex diameter of about 100 nm [14]. Fibres are non-covalently attached to each pentameric penton base at the vertices [15, 16]. Typically, one fibre protrudes from each pentameric vertex. However, either two distinct or two or three identical fibres protruding from each vertex have also been reported [17, 18]. Adenovirus fibres are trimeric proteins, which consists of three different domains: an N-terminal penton base binding domain, a thin central shaft domain and a C-terminal globular fibre head domain [19, 20]. Adenovirus infections are usually initiated by the interaction of their fibre heads with primary receptors, followed by internalization mediated by penton bases interacting with integrins [21].

Adenoviruses from the *Siadenovirus* genus are characterized by a short genome of around 26 kb [12, 13]. Apart from generally conserved adenovirus proteins, five open reading frames potentially encode novel proteins unique to the *Siadenovirus* genus. For instance, at the left end of the genome, a gene encoding a putative sialidase (neuraminidase) was found, which has given the genus its name (*Siadenovirus*). Current members of the *Siadenovirus* genus with published full genome sequences are frog adenovirus 1 (FrAdV-1), turkey adenovirus 3 (TAdV-3), raptor adenovirus 1 (RAdV-1) and South Polar skua adenovirus 1 (SPSAdV-1). TAdV-3 is associated with specific diseases in different hosts: haemorrhagic enteritis in turkey, marble spleen disease in pheasants and splenomegaly in chickens [22, 23]. RAdV-1 was discovered and its genome was sequenced by PCR-based methods, without virus isolation [24, 25]. RAdV-1 was identified as the causative agent of an outbreak of adenoviral disease in the United Kingdom in 2004. RAdV-1 was identified in different birds of prey, including a Harris’s hawk (*Parabuteo unicinctus*), a Bengal eagle owl (*Bubo bengalensis*) and a Verreaux's eagle owl (*Bubo lacteus*) [26]. A single fibre gene has been found in the RAdV-1 genome. It encodes a 464 amino acid protein of which the N-terminal penton-base binding sequence is predicted to contain amino acids 1-75 and the shaft domain residues 76-319, including fifteen triple beta-spiral repeats [20]. The C-terminal fibre head domain is expected to be composed of amino acids 324-464.

Modification of the adenovirus fibre head domain has been performed with the aim of retargeting adenovirus-based vectors to specific cell types [27]. Although many human adenovirus fibre heads are characterized [14], only a few animal adenovirus fibre head structures are known [28–33]. Animal adenovirus fibres may provide novel receptor binding and targeting functions, while humans may also have less pre-existing immunity to them. Recently, the structure of the first fibre head of an adenovirus from the genus *Siadenovirus* was reported, of TAdV-3 [34]. The structure revealed the insertion of a beta-hairpin embracing a neighbouring monomer when compared to known adenovirus fibre head structures. The TAdV-3 fibre head structure was found to bind sialyllactose, which may function as a (co-)receptor. Here we present the structure of the raptor adenovirus 1 fibre head, which does not appear bind sialyllactose, and show that deletion of the beta-hairpin insertion does not significantly affect the stability of siadenovirus fibre head domains.

## Results and discussion

### Expression, purification, crystallization and structure solution of the raptor adenovirus 1 fibre head

Sequence analysis of the putative fibre protein suggested that the fibre head domain likely comprises residues 324 to 464. An expression vector was constructed containing residues 324-464 and the resulting protein was expressed with an N-terminal purification tag (MGSS HHHHHH SSGLV PRGSH MASMT GGQQM GRGSG). Codon analysis of the RAdV-1 fibre gene suggested the presence of a significant amount of rare codons (17 %) and a relatively low GC-content for the fibre head-coding region (just over 30 %). Therefore, the protein was expressed in the *Escherichia coli* Rosetta2(DE3)pLysS strain. The histidine-tagged RAdV-1 fibre head protein was purified by metal affinity chromatography and anion exchange chromatography as described in the Methods section. About 16 mg of purified protein could be obtained per litre of expression culture. The protein was concentrated and stored in a buffer containing L-arginine and glycerol as stabilizing agents.

Vapour diffusion crystallization trials were performed and well-diffracting crystals were obtained after 3–4 days when a solution containing 1.5 M sodium chloride and 10 % (v/v) ethanol was used as a reservoir. A high-quality dataset was collected from one of these crystals and indexed to space group *P*2_1_3, with one protein monomer per asymmetric unit. The structure could be solved by molecular replacement, using a monomer of the TAdV-3 fibre head structure [34] as a search model, despite of them having low sequence identity (19 %; as a rule-of-thumb, 25–30 % sequence identity is usually considered necessary for successful structure solution by molecular replacement). The final refined model contains residues 327-462, plus ordered solvent (water and chloride molecules). No reliable density was observed for the N-terminal purification tag, for residues 324-327 or for the C-terminal threonine and alanine residues (amino acids 463-464), suggesting that these are disordered. Data collection, phasing and refinement statistics are shown in Table 1.

**Table 1:** Crystallographic data and refinement statistics

	Wild-type fibre head	Beta-hairpin deletion
Data collection		
Space group	*P*2_1_3	*P*2_1_3
Cell parameter(s)	81.72	81.40
Wavelength (Å)	0.9795	0.9793
Resolution (Å)^a^	47.2–1.47 (1.49–1.47)^a^	45.0–1.70 (1.73–1.70)
Observed reflections	31365 (1541)	20075 (1045)
Multiplicity	9.9 (8.8)	6.6 (8.6)
Completeness	1.000 (1.000)	100.0 (100.0)
Rmerge^b^	0.035 (0.599)	0.023 (0.695)
<I/sigma(I)>	29.6 (3.2)	374.5 (3.5)
Wilson B (Å^2^)	17.7	29.4
Half dataset corr. coeff. (CC1/2)	0.999 (0.865)	0.998 (0.804)
Refinement		
Resolution range (Å)	45.0–1.47 (1.51–1.47)	45.0–1.70 (1.74–1.70)
No. of reflections used in refinement	29748 (2179)	19065 (1372)
No. of reflections used for Rfree	1586 (127)	994 (72)
R-factor^c^	0.157 (0.222)	0.174 (0.230)
Rfree	0.179 (0.244)	0.198 (0.286)
No. of protein/water/chloride atoms	1122/274/4	978/124/4
Average B for protein/solvent atoms (Å^2^)	23.7/39.0/26.9	38.8/53.1/48.6
Ramachandran plot (favoured/allowed)^d^	0.986/1.000	0.984/1.000
R.m.s.d. bonds (Å) and angles (°)^e^	0.014/1.7	0.012/1.6
PDB code	5FJL	5FLD

^a^Values in parentheses are for the highest resolution bin, where applicable

^b^Rsym = ΣhΣi|Ihi-<Ih>|/ΣhΣi|Ihi|, where Ihi is the intensity of the ith measurement of the same reflection and <Ih> is the mean observed intensity for that reflection

^c^R = Σ||Fobs(hkl) | - |Fcalc(hkl) ||/Σ|Fobs(hkl) |

^d^Determined with MOLPROBITY. The fractions are indicated of residues in favoured and allowed regions of the Ramachandran plot, respectively

^e^Provided by REFMAC (r.m.s.d.: root mean square difference)

### Structure description

**Fig. 1:**
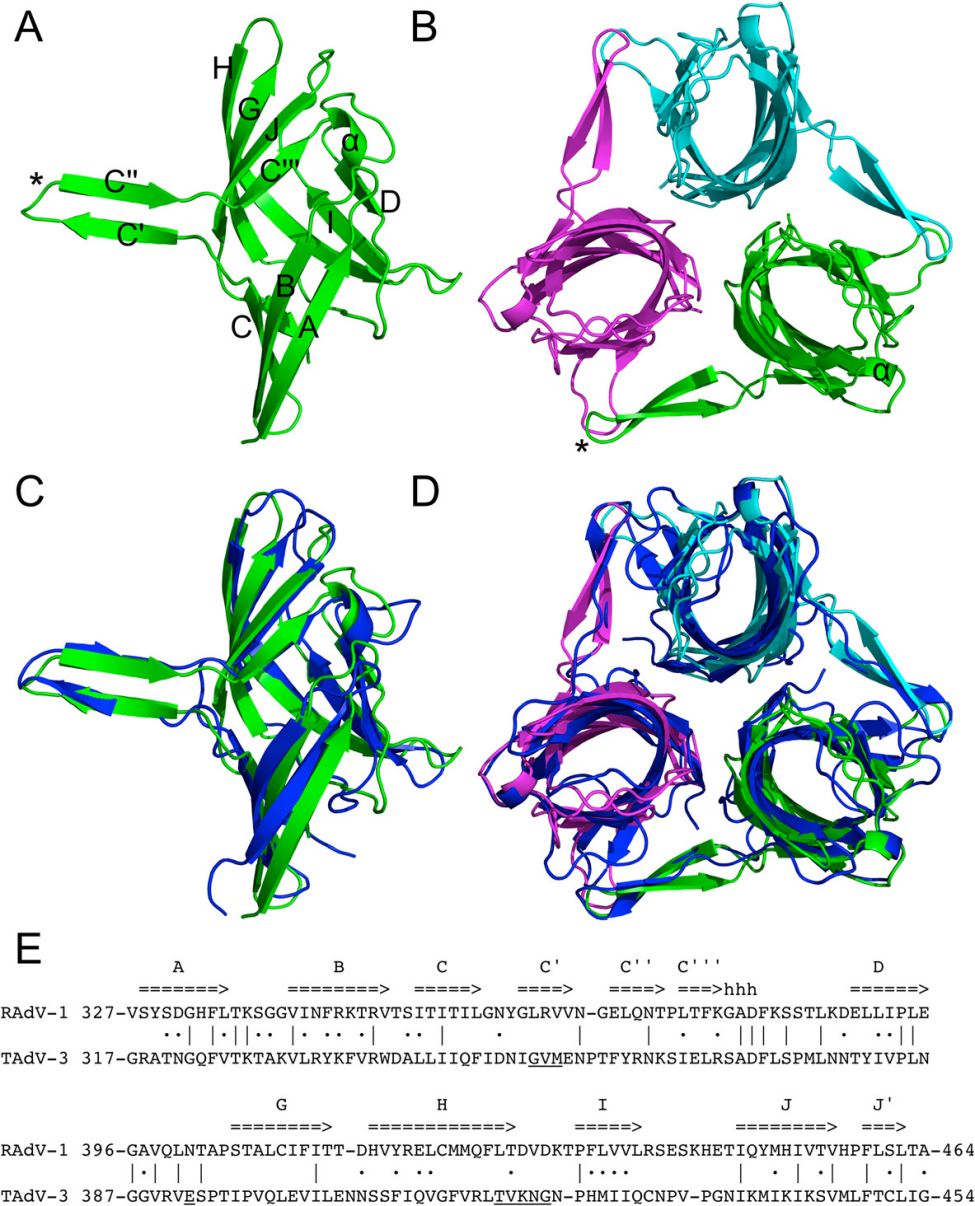
Structure of the RAdV-1 fibre head domain and comparison with the TAdV-3 fibre head. **a** The RAdV-1 fibre head monomer. Beta-strands are labelled (except the J'-strand, which is located behind the A-, B- and C-strands). The beta-hairpin is marked with an asterisk and the short helical region in the CD-loops with an alpha sign. **b** The RAdV-1 fibre head trimer. The three monomers are shown in *green*, *magenta* and *cyan*. The beta-hairpin and helical region are marked as in panel (**a**). **c** The RAdV-1 fibre head monomer (*green*; in the same orientation as in panel **a**) superposed onto the TAdV-3 fibre head monomer (*dark blue*). **d** The RAdV-1 fibre head trimer, coloured as in panel **b**, superposed onto the TAdV-3 fibre head trimer (*dark blue*). **e** Structure-based sequence alignment of the RAdV-1 and TAdV-3 fibre heads. Identical residues are indicated with lines, similar residues with dots. Beta-strands and the short helical region are *highlighted*. The residues that in the TAdV-3 fibre head structure interact with sialyllactose are *underlined*

When looked at from the side, each RAdV-1 fibre head monomer has an elongated shape and is about 5 nm high and 2.5 nm at its widest point with an obtuse triangular longitudinal cross-section (Fig. 1). When viewed from the top, the cross-section is oval and about 2.5 nm long and 1.5 nm wide. A beta-hairpin, made up of residues 359-373, sticks out of each monomer. Together, three crystallographically related monomers form a compact globular trimer. Like the other siadenovirus fibre head structure, that of TAdV-3 [34], the monomer has an ABCJ-GHID beta-sheet topology with kinked C- and J-strands resembling reovirus fibre head structures [35, 36]. The protruding C'C" beta-hairpins, embracing neighbouring monomers, appear to be unique to siadenovirus fibre heads. Despite of the low sequence identity (only ~19 %), the structures are very similar and can be superposed with a root mean square difference (r.m.s.d.) of 1.8 Å^2^ (131 superposed C-alpha atoms, Z-score of 18.6 [37]).

A structural feature present in both siadenovirus fibre head structures is the short alpha-helix in the CD-loop. Apart from being structurally conserved, it is also one of the few regions where the sequence is conserved between the RAdV-1 and TAdV-3 fibre heads, together with the AB-loop and the end of the D-strand and beginning of the DG-loop. These regions are close together in space and may well be important for folding or function of the fibre head. This sequence similarity extends to another avian siadenovirus fibre head sequence known, that of South Polar skua adenovirus fibre head [38], but not to the frog adenovirus 1 fibre head, the fourth siadenovirus fibre head sequence known [39].

**Fig. 2:**
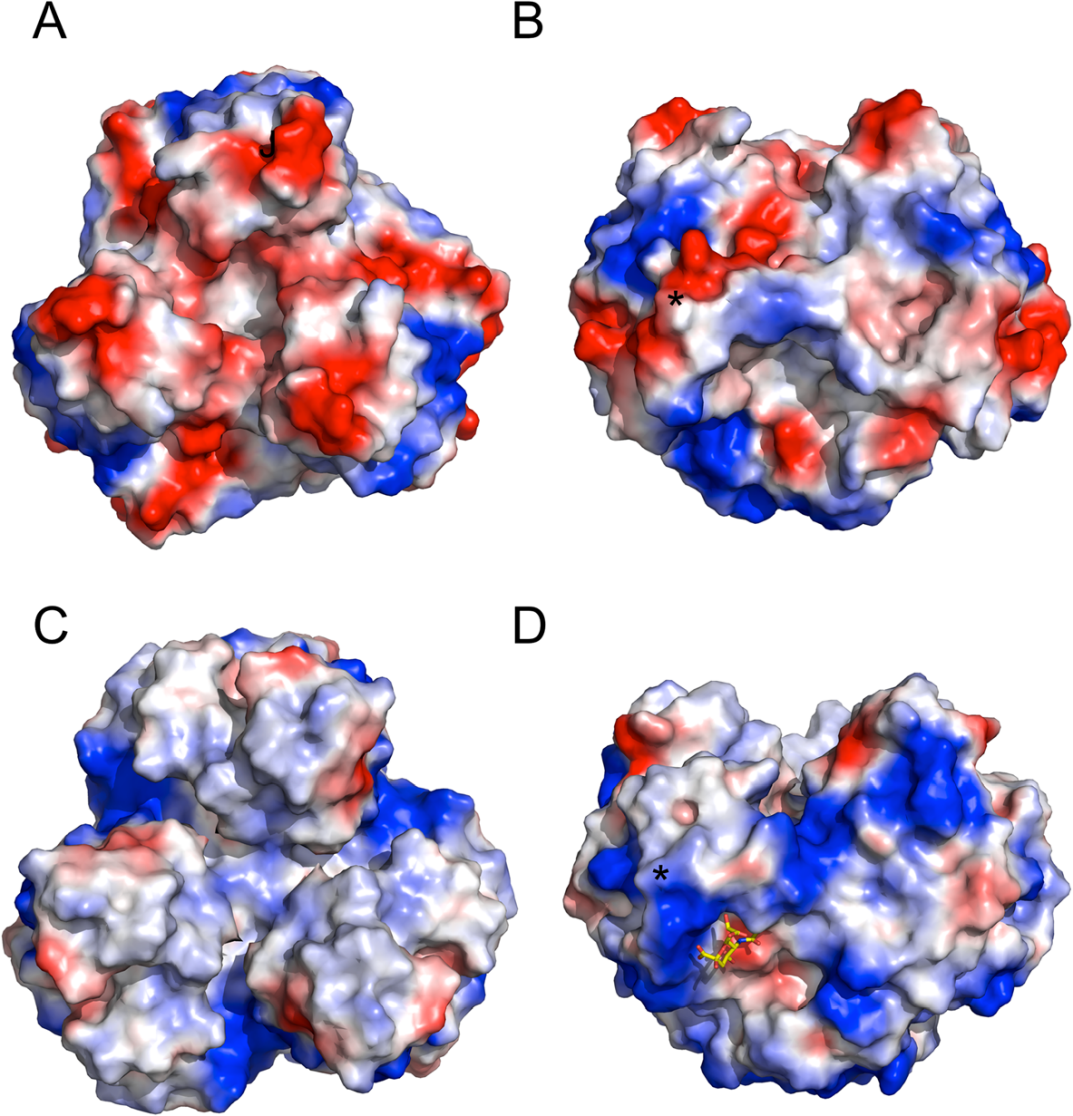
Electrostatic surface potential of the RAdV-1 and TAdV-3 fibre head domains. **a** The RAdV-1 fibre head viewed from the top. **b** The RAdV-1 fibre head monomer viewed from the side. The end of the beta-hairpin is marked with an asterisk. **c** The TAdV-3 fibre head (PDB entry 4D62) viewed from the top. **d** The TAdV-3 fibre head viewed from the side. The end of the beta-hairpin is marked with an asterisk and the 2,3-sialyllactose ligand is shown in stick representation (the ligand is hidden when viewed from the top)

Apart from the similarities between the RAdV-1 and TAdV-3 fibre head structures, some differences are also observed. The calculated surface charge distribution is very distinct between the two fibre heads (Fig. 2). Mainly positively charged patches are observed for the TAdV-3 fibre head, while both negatively and positively charged patches are present in the RAdV-1 fibre head. The electrostatic potential surface charges showed that strong negatively charged patches are observed at the top surface of the RAdV-1 fibre head, while negatively and positively charged patches are found on the sides. The mainly positively charged surface of the TAdV-3 fibre head led us to search for carbohydrate ligands, and 2,3- and 2,6-sialyllactose were identified as potential ligands in glycan micro-array experiments and confirmed by NMR spectroscopy, isothermal calorimetry, co-crystallization and site-directed mutagenesis [34]. In the TAdV-3 structure complexed with 2,3-sialyllactose (PDB entry 4D62), the ligand is found wedged between a negatively (Glu392 is an interaction partner) and a positively charged patch (with Lys421 as a confirmed interaction residue). In the RAdV-1 structure, these charges are inverted and none of the interaction partners is conserved (underlined residues in Fig. 1e). Indeed, we could not identify carbohydrate ligands of the RAdV-1 fibre head by glycan micro-array experiments and co-crystallization with 2,3- and 2,6-sialyllactose was unsuccessful. This supports the idea that the binding of the adenovirus fibre heads with sialylated carbohydrates is charge-dependent, which was proposed previously [40]. Our observations implicate that the cell receptor of these two adenoviruses may be different. Alternatively, perhaps both RAdV-1 and TAdV-3 bind the same, as yet unidentified protein receptor, and only TAdV-3 uses sialyllactose as a co-receptor.

### Stability of siadenovirus fibre heads

In the trimeric form, the interaction interface of the RAdV-1 fibre head contains many hydrophobic interactions, with 23 amino acids from each monomer involved, accounting for 17 % of all residues. Four of them are residues from the beta-hairpin arm (which is composed of residues 359-372) interacting with a neighbouring monomer. The basic framework of the trimeric form is also secured by eighteen intermonomer hydrogen bonds (five of these involve the beta-hairpin arm) and intermolecular salt bridges between Arg419 of one monomer and Asp388/Glu389 pairs of a neighbouring monomer. This compares to the TAdV-3 fibre head trimer, with twenty amino acids (15 % of all residues) involved in hydrophobic interactions (three from the beta-hairpin arm), sixteen intermonomer hydrogen bonds (six involving the beta-hairpin arm) and an intermolecular salt bridge between Arg318 and Asp340.

**Fig. 3:**
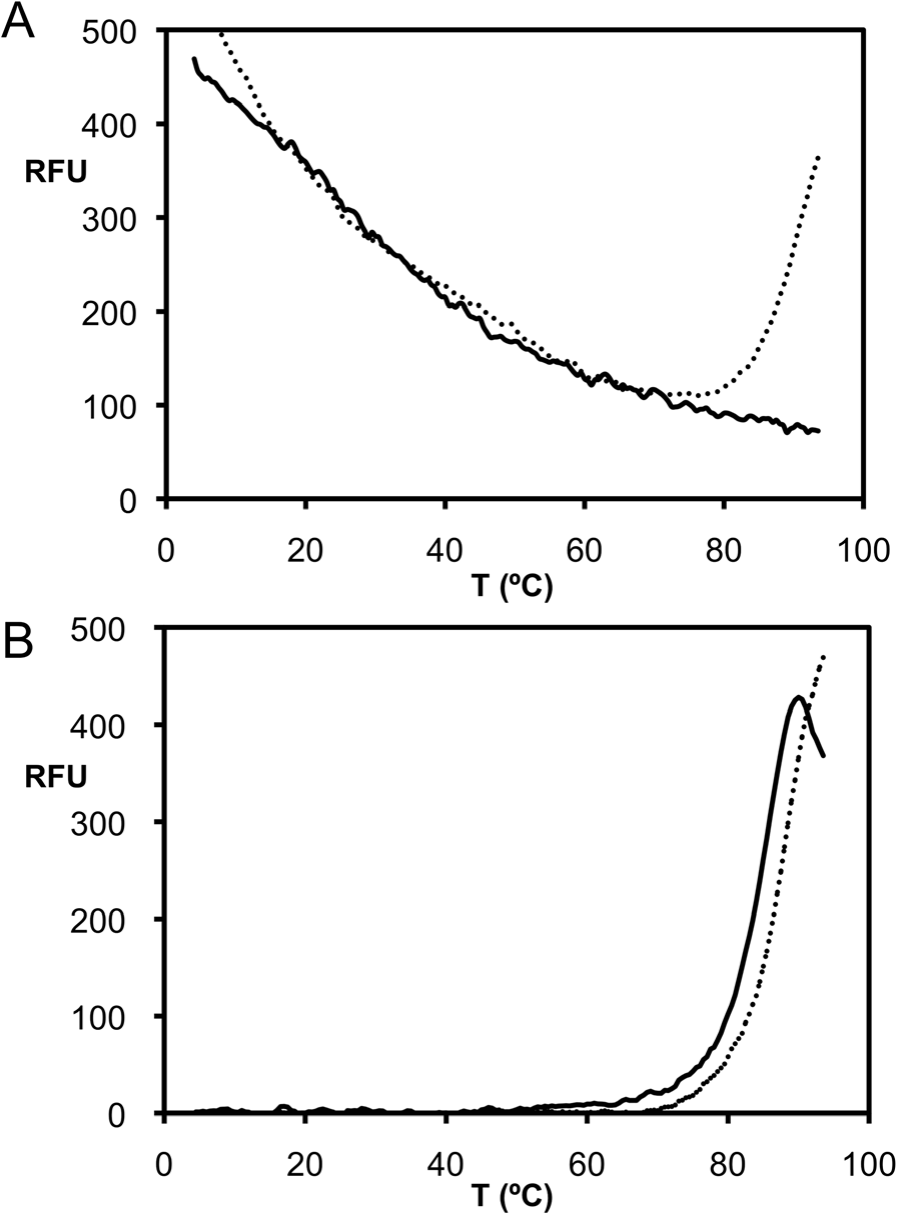
Thermal stability of siadenovirus fibre heads. **a** Thermofluor protein denaturation assay of the RAdV-1 fibre head (with beta-hairpin, *continuous line*) and RAdV-1 fibre head from which the beta-hairpin arm residues 359-373 have been deleted (*dotted line*). **b** Thermofluor protein denaturation assay of the TAdV-3 fibre head (with beta-hairpin, *continuous line*) and TAdV-3 fibre head from which the beta-hairpin arm residues 349-364 have been deleted (*dotted line*)

The RAdV-1 fibre head trimer has a solvent accessible surface area of 16.5 × 10^3^ Å^2^, with 8.1 × 10^3^ Å^2^ of buried surface; this means that 33 % of the total surface gets buried upon trimer formation. The calculated dissociation energy (ΔG_diss_) is 70 kcal/mol, which indicates that the fibre head is very stable. This observation is consistent with the thermal denaturation assay results, in which no denaturation was observed at up 94 °C (continuous line in Fig. 3a). In comparison, the TAdV-3 fibre head trimer has a solvent accessible surface area of 16.8 × 10^3^ Å^2^, with 7.4 × 10^3^ Å^2^ of buried surface; this means that 31 % of the total surface gets buried upon trimer formation; i.e., very similar to the RAdV-1 fibre head. However, the calculated dissociation energy (ΔG_diss_) of the TAdV-3 fibre head is 35 kcal/mol, only half that of the RAdV-1 fibre head. In a thermal denaturation assay (continuous line in Fig. 3b), the unfolding temperature was estimated to be about 86 °C [34], so the TAdV-3 fibre head appears to be less stable than the RAdV-1 fibre head, although it is still a very sturdy protein.

### Deletion of the protruding beta-hairpin

For investigation of the impact of the beta-hairpin on the structure and stability of siadenovirus fibre heads, deletion mutagenesis was performed. The beta-hairpin arms of the RAdV-1 and TAdV-3 fibre heads (residues 359-NYGLR VVNGE LQNTP-373 and 349-DNIGV IENPT FYRNK S-364, respectively) were replaced by a short fragment containing two amino acids (EF and VD in the structure of RAdV-1 and TAdV-3 fibre heads, respectively). About 16 mg and 13 mg of mutated fibre head protein from RAdV-1 and TAdV-3 could be obtained per each litre of bacterial culture, respectively. Mutated fibre head proteins were concentrated up to 10 mg/ml in the same buffer as for their native counterparts. Well-diffracting crystals of the RAdV-1 fibre head beta-hairpin deletion mutant were obtained when 0.1 M 2-[4-(2-hydroxyethyl)piperazin-1-yl]ethanesulfonic acid-NaOH pH 7.5, 20 % (v/v) Jeffamine M-600 (O-(2-Aminopropyl)-O'-(2-methoxyethyl) polypropylene glycol 500) was used as a reservoir solution. Data to 1.7 Å resolution was collected from one of the crystals and processed. This structure could be solved by difference Fourier synthesis and directly refined, as the crystal form was isomorphous to the non-mutated fibre head. Unfortunately, crystals of the TAdV-3 fibre head with the beta-hairpin arm removed could not be obtained.

**Fig. 4:**
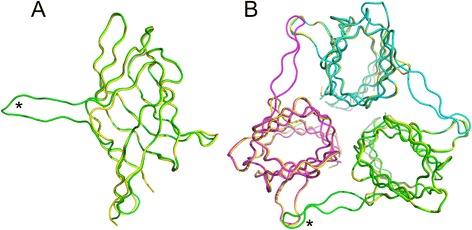
Superposition of the native and mutant RAdV-1 fibre head domain structures. **a** The RAdV-1 fibre head monomer in native (with the beta-hairpin arm; *green*) and mutant form (without the beta-hairpin arm; *yellow*), viewed from the side. **b** The RAdV-1 fibre head trimer in native (with the beta-hairpin arm; monomer in *green*, *magenta* and *cyan*) and mutant form (without the beta-hairpin arm; in *yellow*), viewed from the top. The end of the beta-hairpin of monomer A is marked with an asterisk in both panels

Structure superposition of the native RAdV-1 fibre head and the beta-hairpin arm deletion mutant showed that the main architecture is highly conserved (Fig. 4), apart from the mutation introduced. The beta-hairpin arm is replaced by a short loop and the conformations of the BC- and HI-loops are slightly affected. In the case of the HI-loop this can be explained by the fact that in the trimer, it is close to the end of the beta-hairpin arm of a neighbouring monomer. In the trimeric form, the interaction interface of the mutant RAdV-1 fibre head now only contains hydrophobic interactions involving fourteen amino acids from each monomer, accounting for 11 % of all residues. Only seven intermonomer hydrogen bonds are present, although the intermolecular salt bridge is conserved. The mutated RAdV-1 fibre head trimer has a solvent accessible surface area of 16.9 × 10^3^ Å^2^, with 5.9 × 10^3^ Å^2^ of buried surface, which means that now only 25 % (instead of 33 %) of the total surface gets buried upon trimer formation. The calculated dissociation energy (ΔG_diss_) is 55 kcal/mol instead of 70 kcal/mol.

The thermal stability of the mutated RAdV-1 fibre head protein was assessed by a thermofluor experiment and compared to the native versions (Fig. 3). Deletion of the beta-hairpin appears to destabilize the structure somewhat, because now some unfolding is observed at high temperature (dotted line in Fig. 3a). The melting temperature of the deleted mutant was estimated to be about 90 °C, although unfolding was not complete at the maximum temperature at 94 °C reached in the experiment. The somewhat lower unfolding temperature can be explained by the loss of hydrophobic interaction surface and some of the intermolecular hydrogen bonds made by the beta-hairpin arm, and is consistent with the assembly stability prediction. In the case of the TAdV-3 fibre head, deletion of the beta-hairpin appears to lead to a slight increase in stability, from an unfolding temperature of 86 °C to 89 °C (Fig. 3b). In this case, perhaps the mutant is more compact than the native protein. This could be caused by a certain flexibility of the beta-hairpin arm, a flexibility that may be absent in the RAdV-1 fibre head.

## Conclusion

The high-resolution structure of RAdV-1 fibre head is the second known structure of a siadenovirus fibre head domain. The structure shows that the siadenovirus fibre head structure is conserved, including the alpha-helix in the CD-loop as well as the beta-hairpin insertion in the C-strand. Differences in the predicted surface charge suggest that RAdV-1 uses a different natural receptor for cell attachment than TAdV-3. Deletion of the beta-hairpin arm shows little impact on the structure of the RAdV-1 fibre head, although it slightly destabilizes the very sturdy structure. In contrast, deletion of the beta-hairpin arm in the TAdV-3 fibre head stabilizes its structure somewhat. Further information on the infection mechanism of RAdV-1 may come from infection studies, but for that the virus must first be isolated and a suitable cell culture system be established.

## Methods

### Cloning, expression and purification

A DNA fragment coding for residues 324-464 of the fibre protein was amplified by PCR from the complete DNA genome (GenBank accession number NC_015455.1) and cloned into the expression vector pET28a(+) (Novagen, Merck, Darmstadt, Germany), previously digested with the restriction enzymes *Bam*HI and *Xho*I. The resulting expression vector was called pET28-RAdVFib(324-464). Codon and GC-content analysis were performed using the OptimumGene tool from GenScript (Piscataway NJ, U.S.A.).

For protein expression, *Escherichia coli* strain Rosetta2(DE3)plysS (Novagen, Merck Millipore, Madrid, Spain) was transformed with pET28-RAdVFib(324-464) and grown aerobically at 37 °C in growth medium containing 2 % (w/v) tryptone (pancreatic digest of casein), 1 % (w/v) yeast extract and 20 mM glucose. When the optical density at 600 nm reached 0.5–0.8, the culture was cooled on ice for 30 min and isopropyl-beta-D-1-thiogalactopyranoside was added to a final concentration of 0.5 mM for induction of protein expression. The culture was then incubated overnight at 16 °C with shaking. Cells from two litres of culture were harvested by centrifugation (10 min at 5,000 × g), re-suspended in buffer A (10 mM Tris–HCl pH 7.5, 0.5 M sodium chloride, 10 % (v/v) glycerol) plus 20 mM imidazole and stored at −20 °C. After thawing, cells were lysed by two passes through a French press at about 7 MPa. Cell rests were removed by centrifugation for 30 min at 20,000 × g.

For purification, two ml of nickel-nitrilotriacetic acid resin slurry (Jena Bioscience, Jena, Germany) was added to the protein-containing supernatant and incubated with occasional gentle shaking for 30 min on ice. The resin was then transferred to a column and washed with 30 ml of buffer A with 20 mM imidazole. RAdV-1 fibre head was eluted using a step-gradient of imidazole in buffer A (50 mM, 100 mM, 250 mM and 500 mM imidazole; steps of 5 ml). After analysis by denaturing gel electrophoresis, fractions containing 100 mM, 250 mM and 500 mM imidazole were pooled, dialysed against 10 mM bicine-NaOH pH 9.0 and loaded onto a Resource Q6 column (GE-Healthcare Biosciences, Uppsala, Sweden) equilibrated in the same buffer. The protein was eluted with a linear gradient of 0–1 M sodium chloride in 10 mM bicine-NaOH pH 9.0. Fractions containing pure protein were concentrated to 26 mg/ml using an Amicon Ultra concentrator with a molecular weight cut-off of 10 kDa (Millipore, Madrid, Spain). Three washes with protein storage buffer (10 ml of 10 mM bicine-NaOH pH 9.0, 50 mM magnesium chloride, 5 % (v/v) glycerol and 5 mM L-arginine) were applied. The sample was stored at 4 °C prior to crystallization trials.

### Mutagenesis

The pET28-RAdVFib(324-464) plasmid was used as a DNA template for PCR-based mutagenesis, using the QuikChange procedure (Agilent Technologies, Waldbronn, Germany). For the beta-hairpin deletion mutation, a pair of primers was designed with the insertion of an *Eco*RI restriction site at the 5' end (primers 5'-TAT GAA TTC CTT ACA TTT AAA GGG GCA GAT-3' and 5'-ATA TAG AAT TCT CCA AGG ATT GTA ATC TTA-3'). A standard PCR procedure was performed to obtain a linear DNA product, which was digested with the restriction enzyme *Eco*RI before being self-circulated by T4-DNA ligase and transformed into *Escherichia coli* Top 10. Mutagenesis was confirmed by DNA sequence analysis (Secugen, Madrid, Spain). The protein was produced in the same way as the non-mutated version. The same procedure was performed for beta-hairpin deletion of the TAdV-3 fibre head (using the primers: 5'-CTA TAG TCG ACA TTG AAT TAA GAT CTG CTG ATT TC-3' and 5'-CTA TAG TCG ACT ATA AAC TGT ATG ATT AAC AGA GC-3' with the restriction enzyme SalI) and protein was produced using previously established methods [41].

### Thermal unfolding assay

Thermal unfolding assays [42] were carried out in an iCycler iQ PCR Thermal Cycler (Bio-Rad, Hercules CA, USA) in the presence of the fluorescent dye SYPRO Orange (Life Technologies SA, Madrid, Spain). Reaction volumes of 30 μl were prepared in 200 μl eppendorf tubes, containing 30 μg of protein and 5X SYPRO Orange from the supplied 5000X stock solution. Thermal denaturation curves were obtained by heating the samples from 4 to 94 °C with a ramp rate of 1 °C/min and monitoring the fluorescence at every 0.5 °C increment. The melting temperature T_m_ is defined as the point where the slope of the fluorescence increase is maximal.

### Crystallization, crystallographic data collection and structure solution

The RAdV-1 fibre head proteins were crystallized using the sitting drop vapour diffusion method, using either a robotic setup (Genesis RSP 150 workstation; Tecan, Männedorf, Switzerland) or manually. In either case, 50 μl reservoirs were employed, and drops were prepared containing 0.2 μl of protein sample and 0.2 μl of the respective reservoir solution for robotic setups and 0.6 μl of protein plus 0.6 μl of reservoir solution for manual setups. Crystals were harvested with Litholoops (Molecular Dimensions, Newmarket, England) or Micromounts (Mitegen, Ithaca NY, U.S.A.), transferred to cryo-protection solution (reservoir solution containing 20 % (v/v) glycerol) and flash-cooled in liquid nitrogen.

Crystallographic data were collected at the BL13-XALOC beamline of the ALBA synchrotron [43], integrated using iMosflm [44] and further processed using POINTLESS, AIMLESS and TRUNCATE [45] from the CCP4 suite [46]. Molecular replacement was performed using PHASER [47], using the TAdV-3 fibre head structure (PDB entry 3ZPE) [34] as a search model. The model obtained from PHASER was used as input for automated model building in ARP/WARP [48]. This model was completed using COOT [49] and refined using REFMAC5 [50]. Validation was done with MOLPROBITY [51]. Structure comparisons were performed using the DALI server [37]. Structure figures were made using PYMOL (The PYMOL Molecular Graphics System, Schrödinger, LLC). Protein assembly parameters were calculated using PISA [52]; interaction residues were identified using the PIC server [53].
